# Supplementary material for: Transcriptome-Wide Analysis of Human Chondrocyte Expansion on Synoviocyte Matrix
Source: Cells. 2019 Jan 24;8(2):85. doi: 10.3390/cells8020085 (PMC6406362; doi:10.3390/cells8020085)
Supplement: Supplementary file 1 [file cells-08-00085-s001.zip › 2nd rev supp/S1T1.docx]

A1P: RIN 7.6


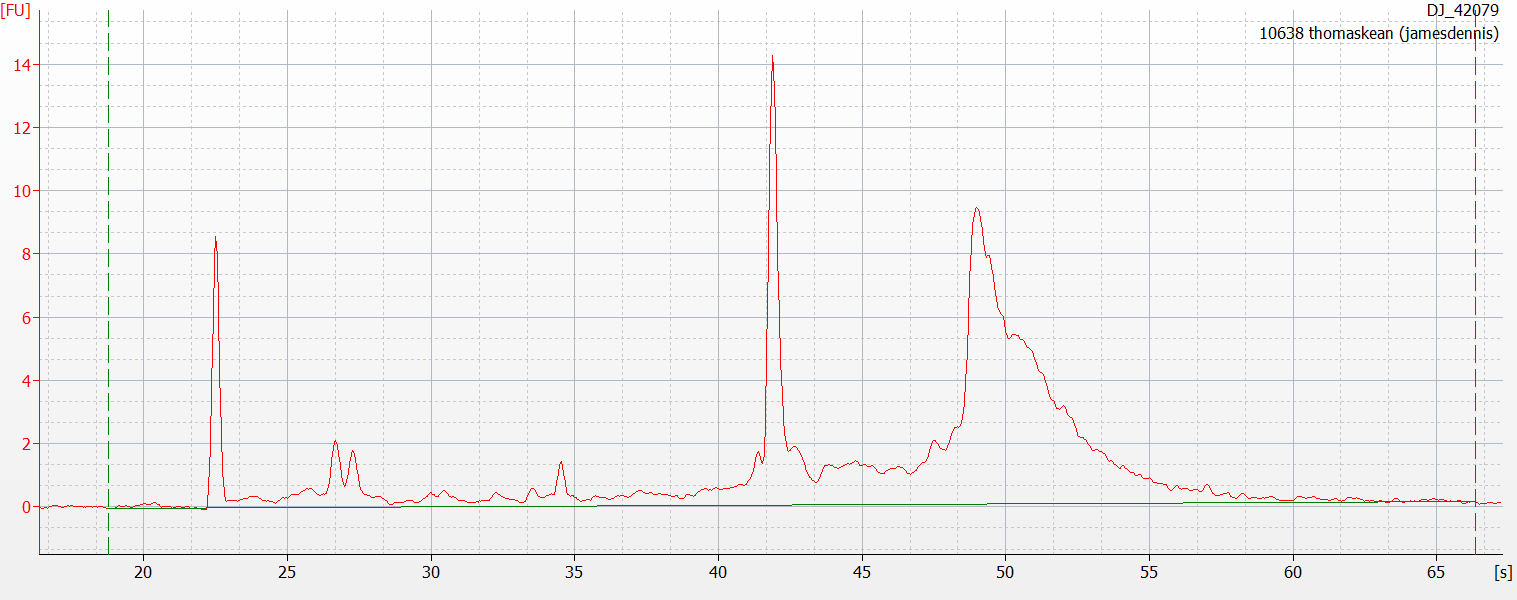


A1S: RIN 9.8


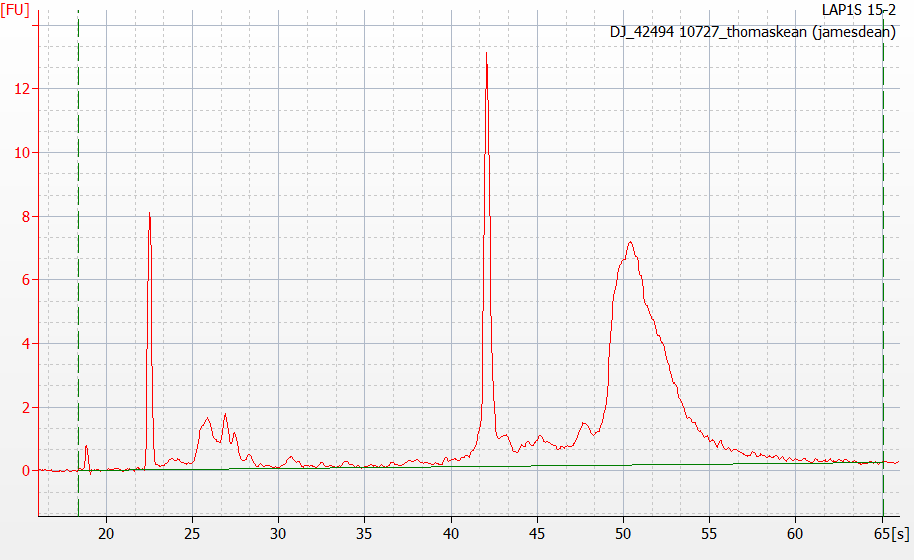


A4P: RIN 9.5


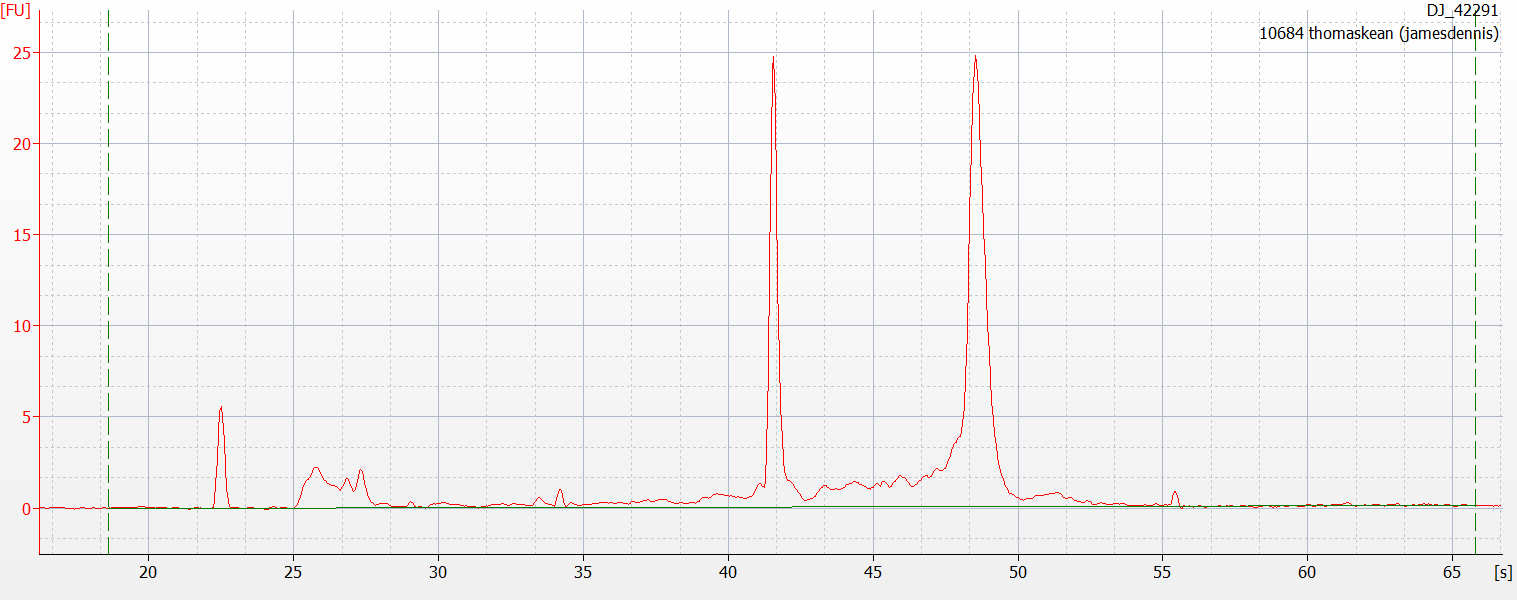


A4S: RIN 9.6


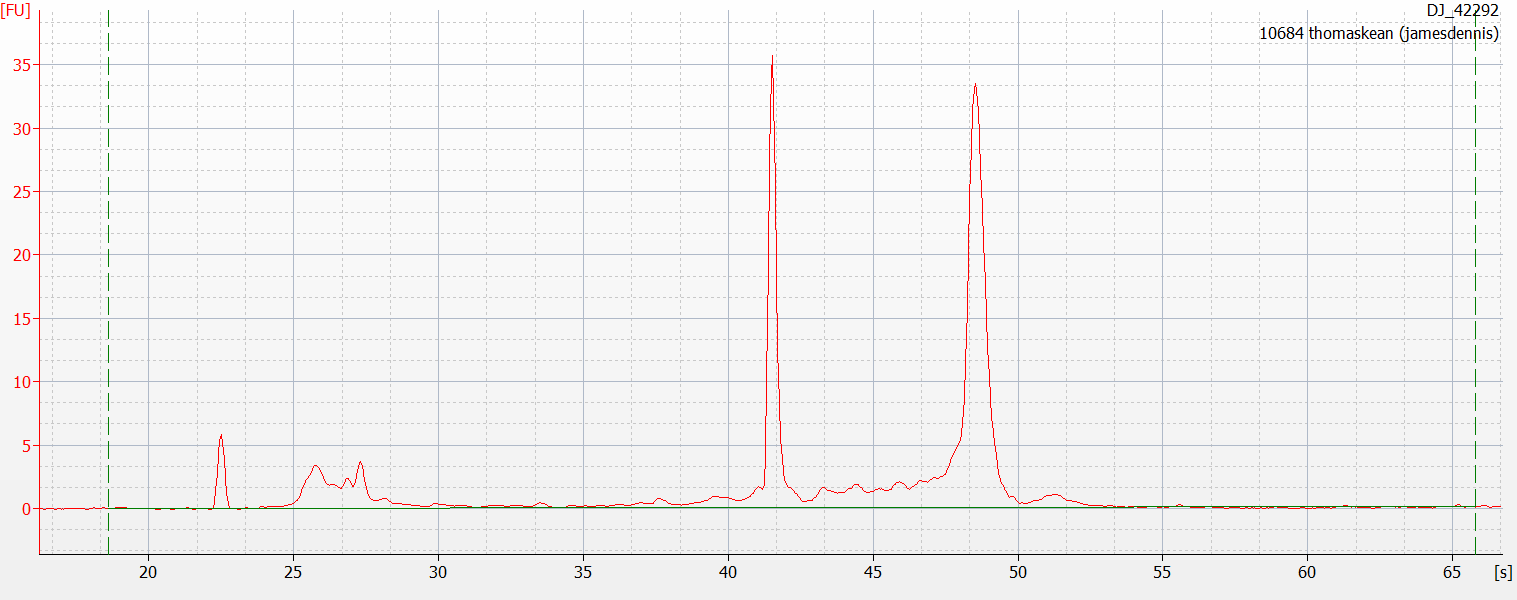


B1P: RIN 7.6


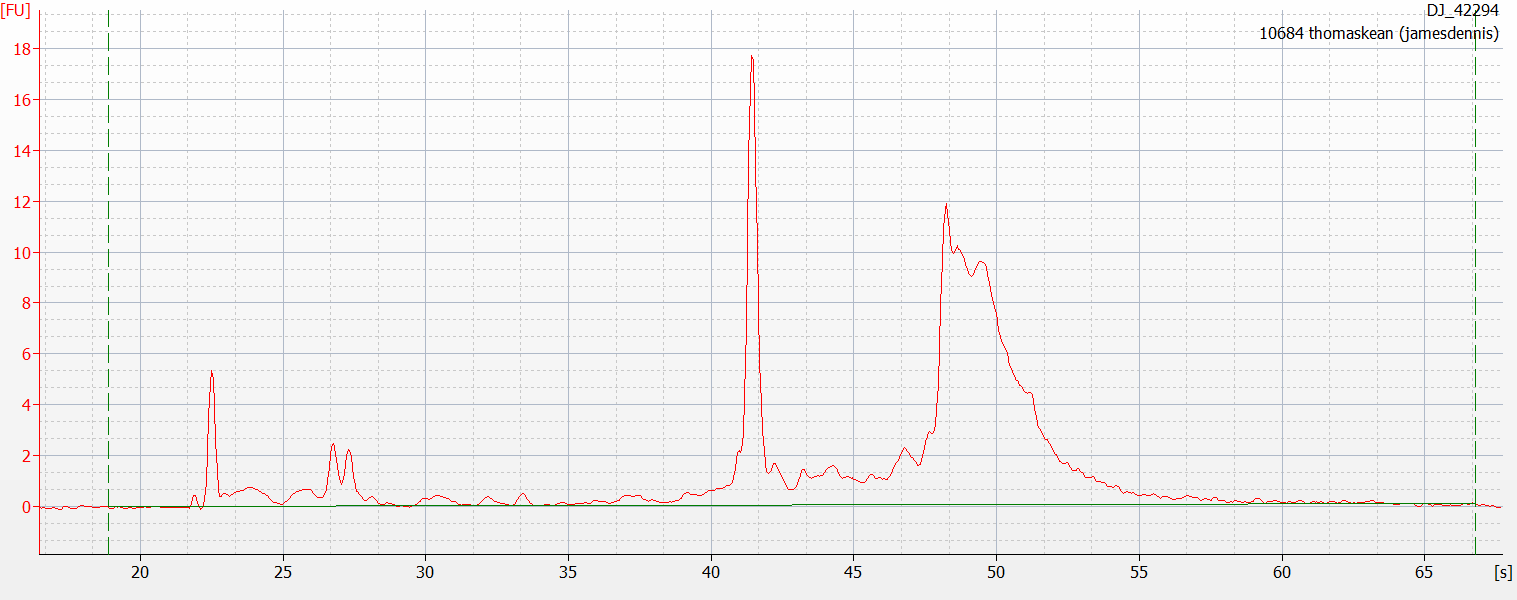


B1S: RIN 8.8


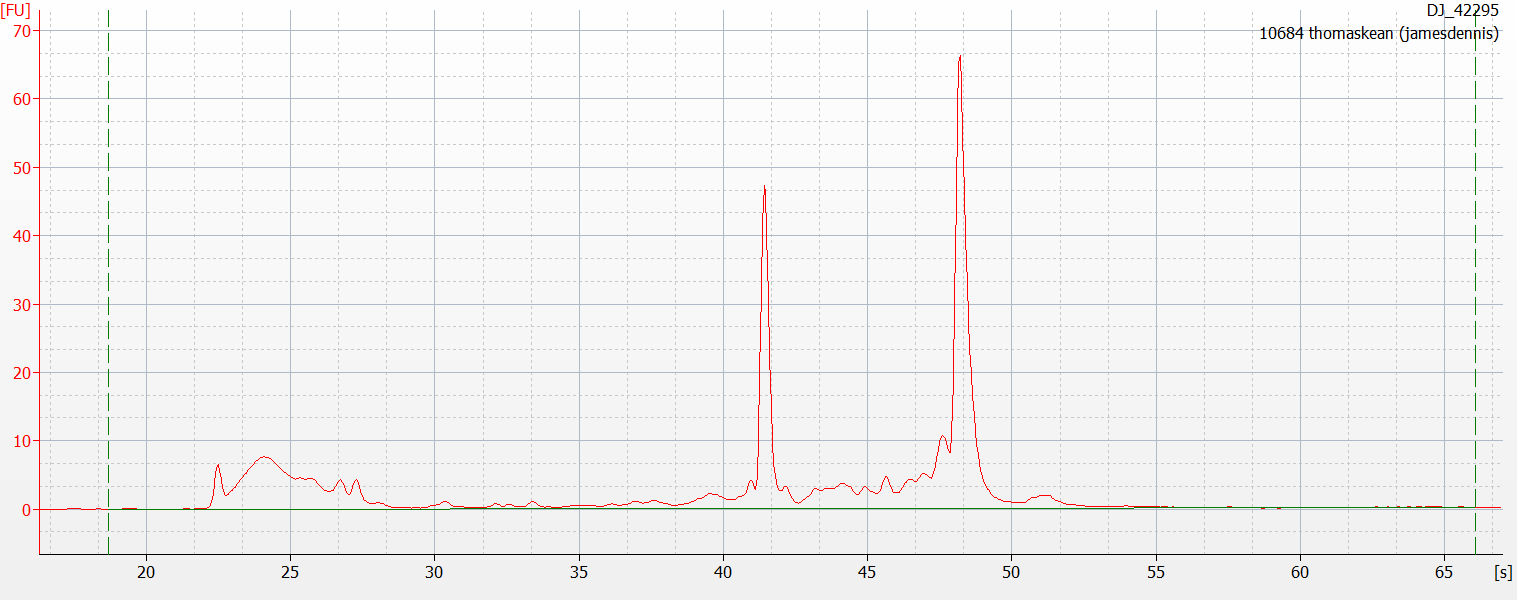


B4P: RIN 9.7


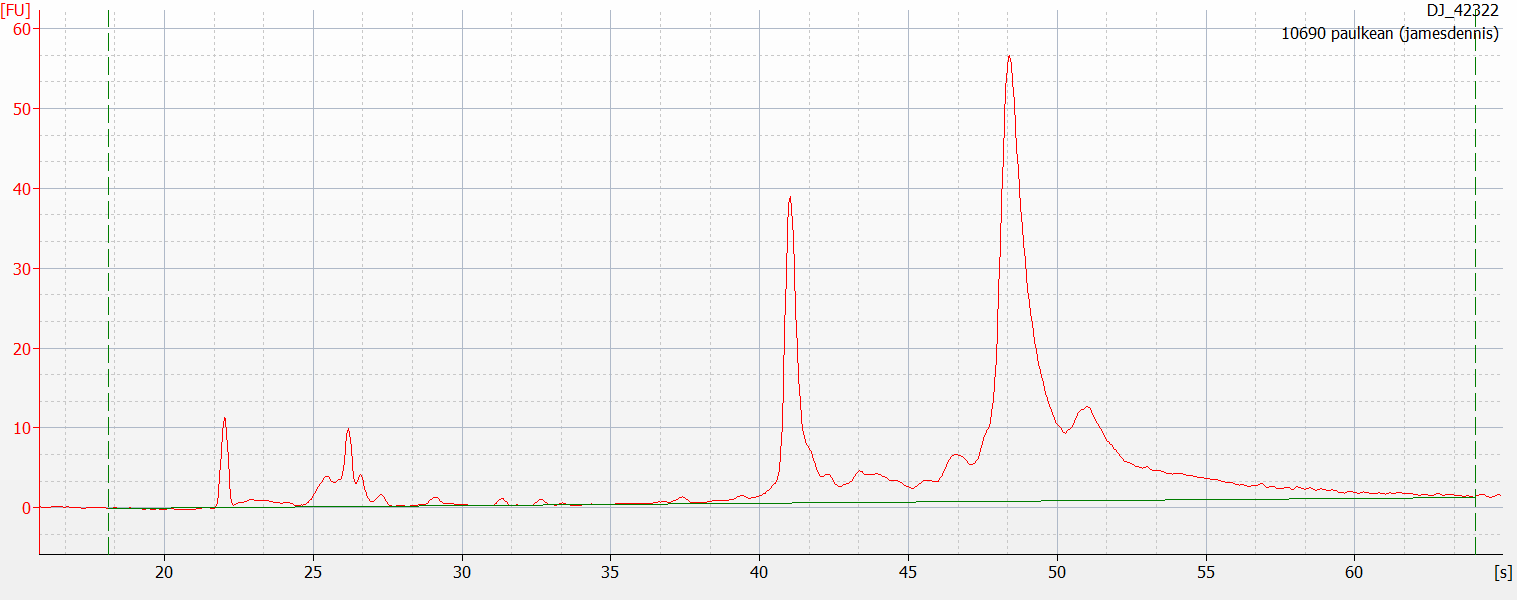


B4S: RIN 9.5


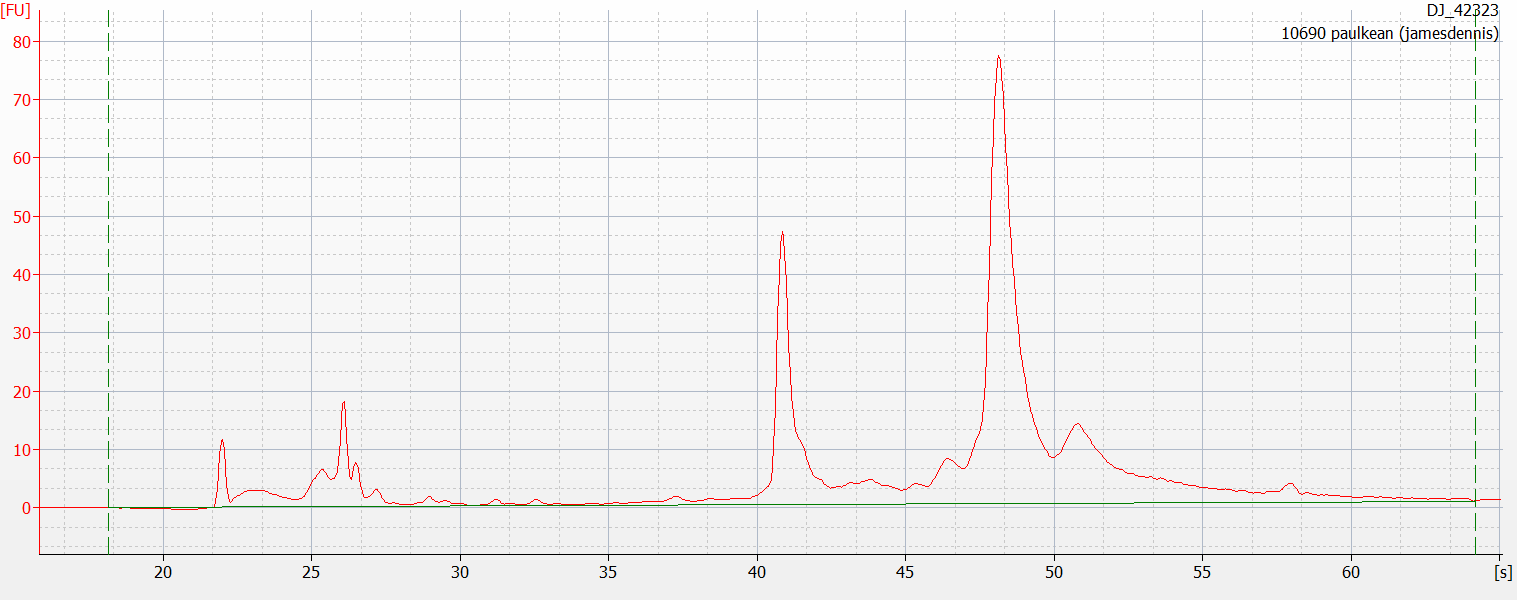


C1P: RIN 9.1


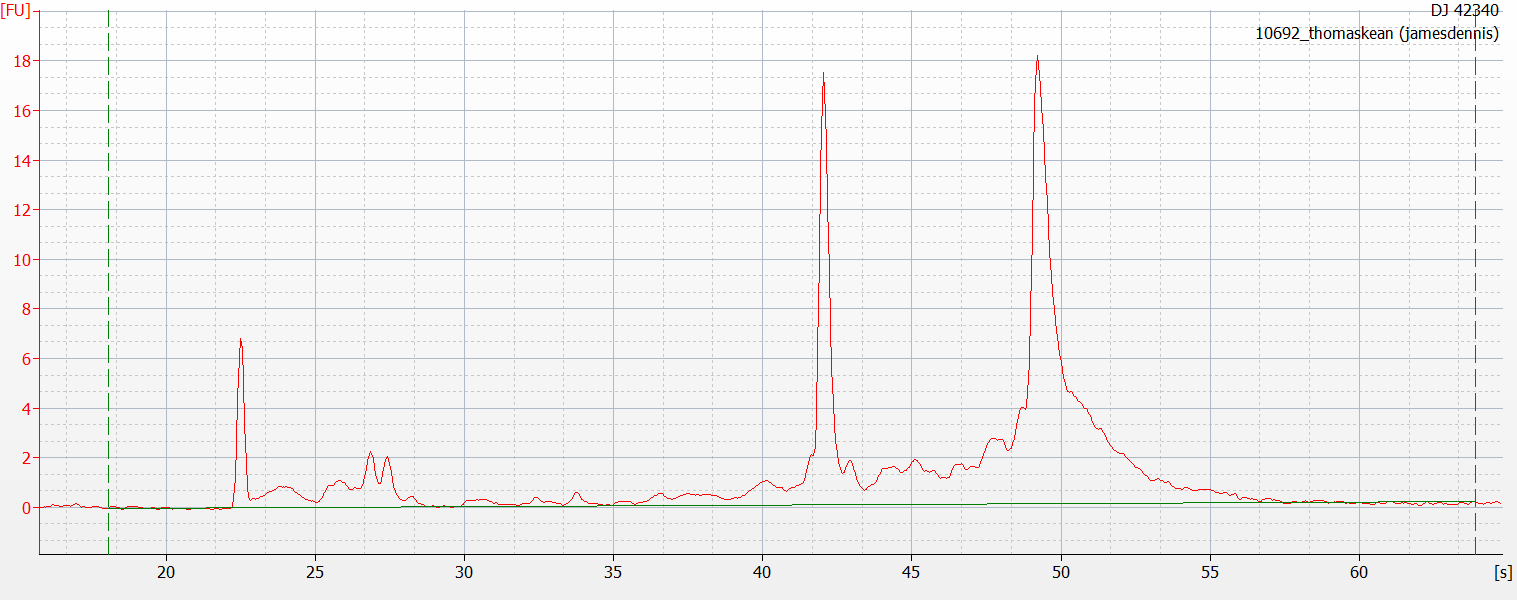


C1S: RIN 8.3


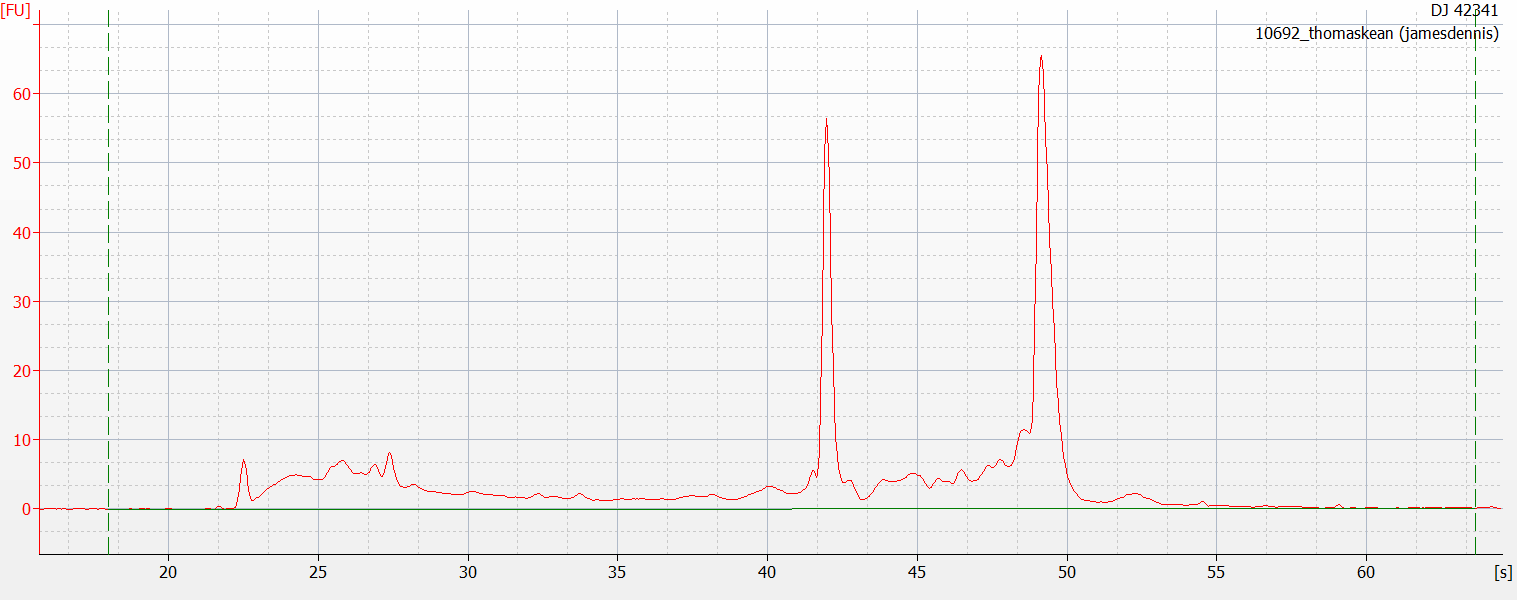


C4P: RIN 9.7


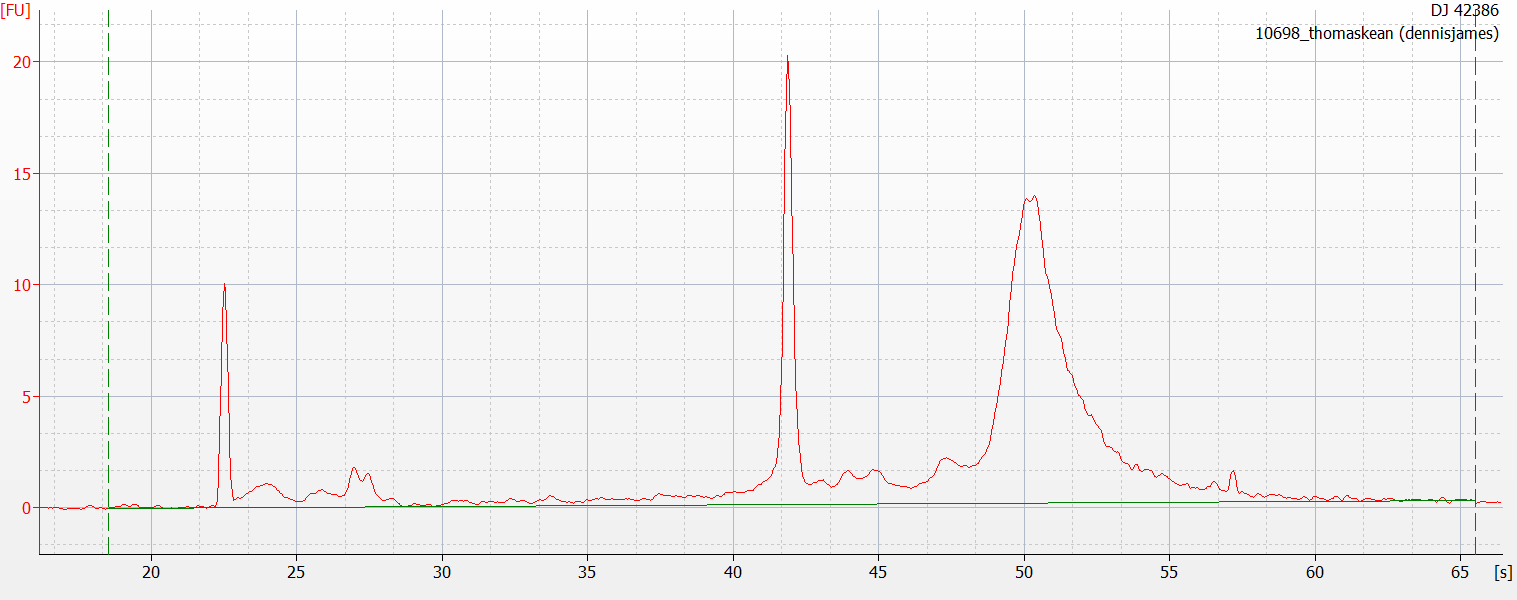


C4S: RIN 8.1


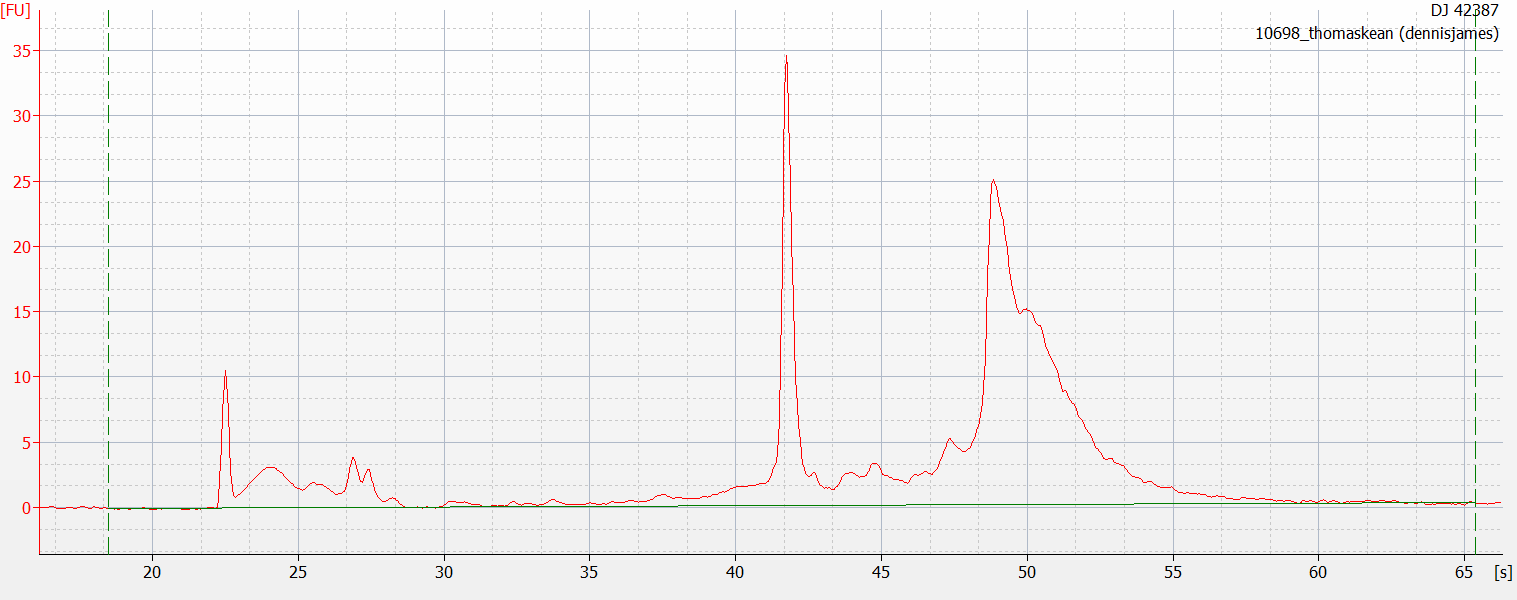


**Table S1.** RNA Analysis Summary

| **Sample** | **RIN** | **ng/ul** | **260/280** | **260/230** |
| --- | --- | --- | --- | --- |
| A1P | 7.6 | 111.03 | 2 | 2.04 |
| A1S | 9.8 | 79.13 | 2.04 | 2.34 |
| A4P | 9.5 | 91.24 | 1.88 | 2.15 |
| A4S | 9.6 | 157.54 | 1.94 | 2.15 |
| B1P | 7.6 | 143.6 | 2 | 2.11 |
| B1S | 8.8 | 247.61 | 2.02 | 2.1 |
| B4P | 9.7 | 84.9 | 1.91 | 2.02 |
| B4S | 9.5 | 171.27 | 1.97 | 2.18 |
| C1P | 9.1 | 122.26 | 1.93 | 1.6 |
| C1S | 8.3 | 288.4 | 1.96 | 2.15 |
| C4P | 9.7 | 101.69 | 1.85 | 1.73 |
| C4S | 8.1 | 164.88 | 1.88 | 1.95 |
