# Supplementary figures and images for: Transcriptome-Wide Analysis of Human Chondrocyte Expansion on Synoviocyte Matrix
Source: Cells. 2019 Jan 24;8(2):85. doi: 10.3390/cells8020085 (PMC6406362; doi:10.3390/cells8020085)

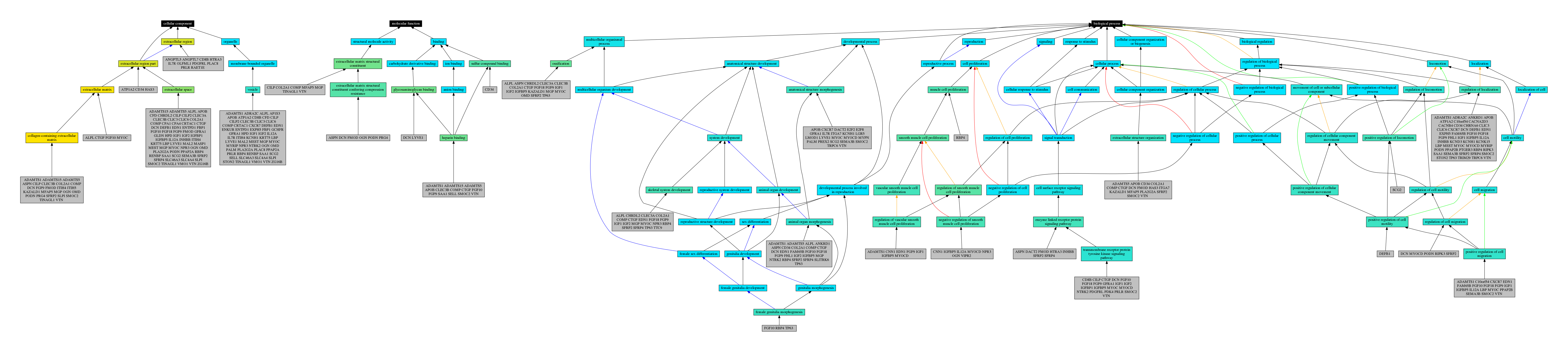

Supplement: Supplementary file 1 [file cells-08-00085-s001.zip › 2nd rev supp/S6.png]

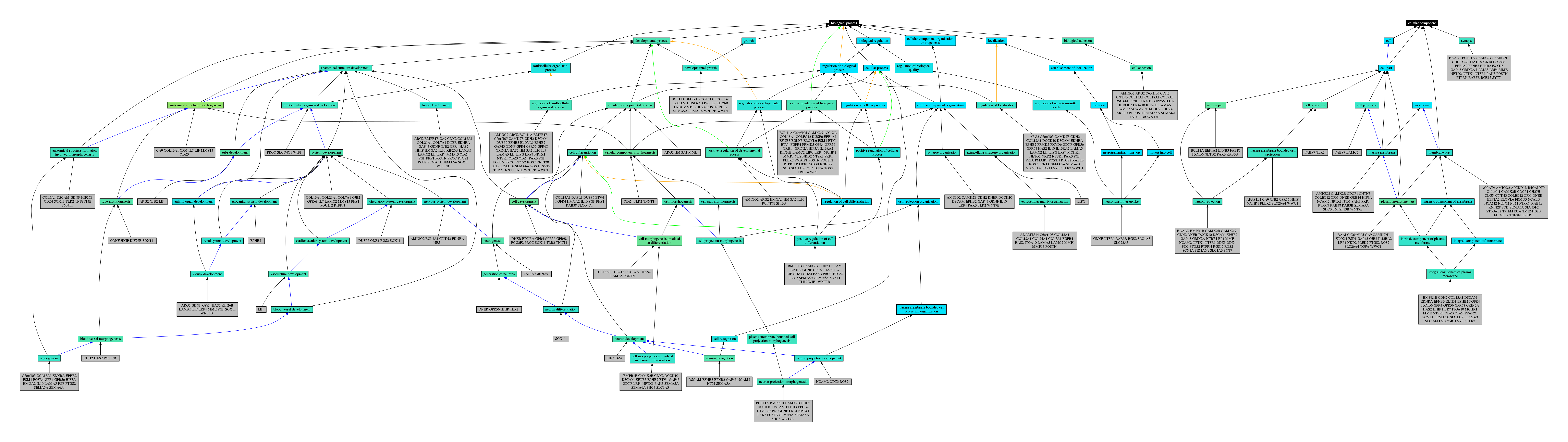

Supplement: Supplementary file 1 [file cells-08-00085-s001.zip › 2nd rev supp/S7.png]

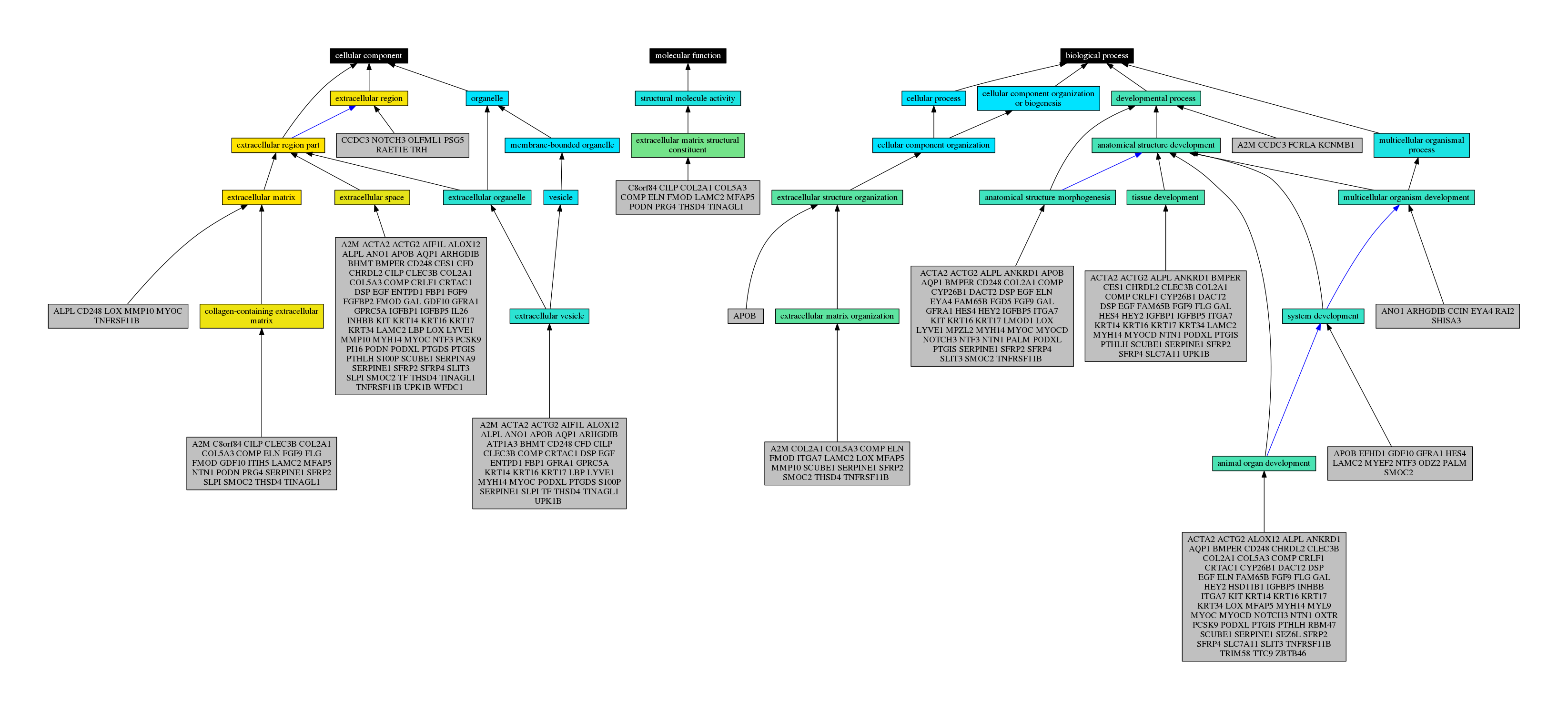

Supplement: Supplementary file 1 [file cells-08-00085-s001.zip › 2nd rev supp/S8.png]

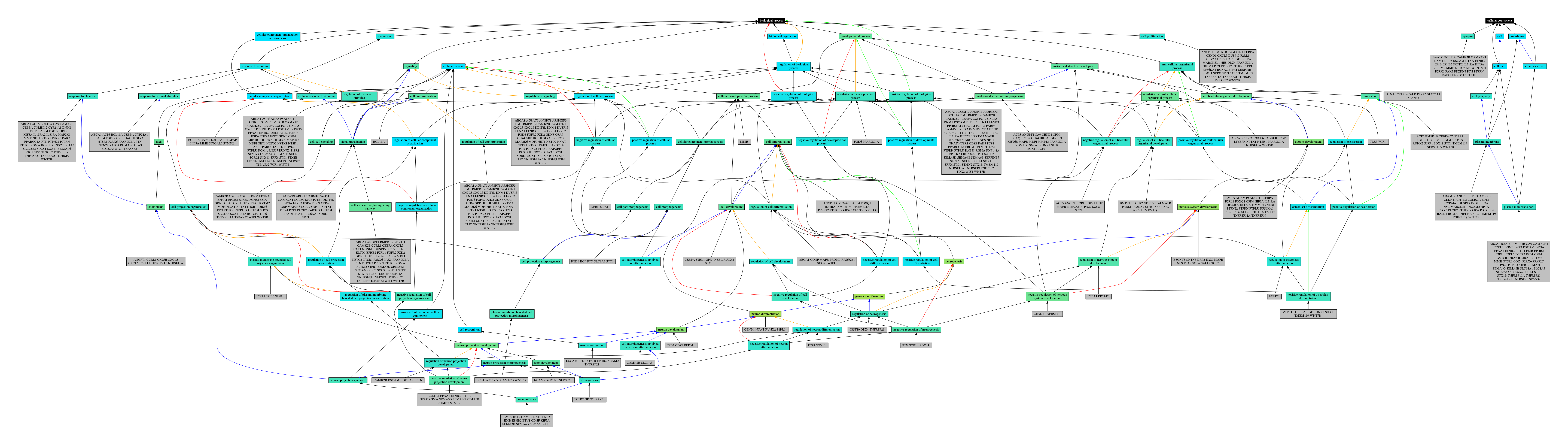

Supplement: Supplementary file 1 [file cells-08-00085-s001.zip › 2nd rev supp/S9.png]
